# Farmland hydrological cycle under agroforestry systems and efficient use of water resources in the karst desertification environment

**DOI:** 10.1016/j.heliyon.2024.e35506

**Published:** 2024-07-31

**Authors:** Qinglin Wu, Lan Wang

**Affiliations:** a School of Karst, Guizhou Normal University, State Engineering Technology Institute for Karst Desertification Control, Guiyang 550001, China; b School of Foreign Languages, Guizhou Normal University, Guiyang 550001, China

**Keywords:** Agroforestry, Conversion mechanism, Efficient utilization of water resources, Five water, Karst

## Abstract

In karst desertification (KD) regions, surface water (SW) easily enters underground through pore fissures and sinkholes despite the presence of abundant precipitation. Such regions have a typical distribution of “soil above and water below”, and, thus, the unique “karst drought” occurs. Hence, an urgent and primary problem in combating KD is to reach highly efficient utilization of water resources in these regions. We selected three karst research areas with different levels of karst desertification and different geomorphic types. By monitoring the storage and transformation of five types of water in the agroforestry system—precipitation, SW, groundwater (GW), soil water (SoW), plant water (PW), the following results were obtained: (1) In KD regions, a positive correlation was found among available precipitation, rainfall, and land evapotranspiration (LE), and LE was approximately equivalent to soil evaporation. (2) To varying degrees, agroforestry brings ecological benefits, including reducing surface runoff, increasing soil infiltration, lowering the transpiration rate, and reducing soil evaporation, thus achieving efficient use of water resources. (3) From 100 % rainfall, the transformation rates of SW, GW, PW, and SoW reached 0.14–12.71 %, 9.43–30.20 %, 9.79–49.97 %, and 40.72–82.58 %, respectively, and SoW showed a larger reserve than the other three types. (4) Drought stress contributes to the improvement of water use efficiency (WUE). Affected by drought stress, WUE was found to be the highest in a medium-intensity karst desertification environment. The transformation mechanisms of the five types of water observed in the agroforestry system provide a reference for efficient utilization of water resources in KD regions as well as theoretical support for addressing karst drought. They are also essential in helping to advance the ecological derivative industry, boosting the economy in karst mountainous areas, and controlling karst desertification.

## 1 Introduction

Karst is a term used to describe a special type of landscape that contains caves and extensive underground water systems. Carbonate rocks crop out over approximately 10 % of the world's land area, and roughly 20–25 % of the global population depends largely or entirely on ground water (GW) in karst regions [1]. China has the largest and most widely distributed karst area, accounting for approximately one-third of the total national land area (3.4 million km^2^), mainly in Southwest China [2,3]. Karst areas are the world's primary ecologically fragile zones and have great significance to the academic community [4]. Karst desertification (KD) is the process of land degradation caused by a fragile ecological environment and unreasonable anthropogenic activities, occurring as soil erosion, a sharp drop in vegetation, rock exposure, land deterioration, and other ecological environmental problems [[5], [6], [7]]. It has been seen as one of the three major ecological disasters in China (drought in the northwest, soil erosion in the Loess Plateau, and karst desertification in karst areas) [8,9]. In karst desertification (KD) regions, despite abundant rainfall, the high soil infiltration rate lead to rainwater easily infiltrating into the subsurface, resulting in a shortage of surface water (SW) but abundant GW resources. GW is usually deeply buried, making it difficult to access [10], thus the unique “karst drought” occurs [11,12]. Karst drought is agricultural drought (a lasting meteorological drought that can develop quickly into a soil moisture shortage, resulting in reduced agricultural production) [13]. It is one of the most important factors limiting the growth, photosynthesis, and distribution of plants in karst habitats [14]. Frequently occurring in the karst areas of southwest China [15], karst drought can be resisted by improving Water use efciency (WUE). WUE is related to the dry matter mass produced by vegetation consumption per unit mass of water in the ecosystem [16]. WUE of farmland ecosystem depends on the farmland evapotranspiration [[17], [18], [19], [20]]. Compared with crop monoculture, agroforestry increases the surface coverage, effectively reduces soil evaporation, and lowers the inner canopy temperature through shading [21]. Accordingly, it can diminish the plant transpiration [22,23], inhibit the ineffective water consumption, and promote the WUE of crops [24]. Also, agroforestry has the advantage that the most crops are planted on the smallest amount of land, by which the crops are increased and the economy is made more profitable. Given the fact that karst region is the rainfed agriculture region, agroforestry shows the superiority of high WUE as well in that more economic benefits can be achieved by consuming the same amount of water.

The term “five water” is proposed to represent the five forms of water that exist in the hydrological cycle [25]. The transformation process of five water forms in a karst area refers to the mutual transformation process among precipitation, ground water (GW), evapotranspiration water, soil water (SoW), and surface water (SW) [26]. Accordingly, the five water forms can be divided into precipitation, SW (surface runoff), GW, SoW (soil moisture content and soil evaporation), and plant water (PW) (transpiration and vegetation interception) [[27], [28], [29]]. Precipitation is the total water resource, of which the available precipitation can be obtained by removing the evapotranspiration. Available precipitation signals the amount of water resources in a region [30]. SoW is the core of the farmland hydrological cycle, closely linking rainfall, SW, GW, and PW [31]. It exists in two forms: storage and conversion. The former refers to the amount of water stored in soil, while the latter indicates how much SoW can be converted into SW through regressive flow, into GW when percolating underground, into the atmosphere through evapotranspiration, and into plants when water is taken up by vegetation. There is a blurry boundary between GW and SoW. Normally, the soil root layer contains SoW, while GW consists of water beneath the root layer of plants and water bodies in conduits and caves. PW consists of water from plant transpiration and vegetation interception. In extremely intensive karst desertification areas, rock fissure water (GW) serves as the main water resource for plants [32]. In this study, the SW was monitored in runoff plots, so it is referred to as runoff. On a farmland scale, the five forms of water are either stored or transformed, forming a hydrological mini-cycle. Water conversion based on the general law of the water cycle is the scientific basis of water resource evaluation and rational utilization and the theoretical basis of water saving research [33]. The law of five-water transformation provides theoretical support for efficient utilization of water resources.

In previous studies on five-water conversion in karst areas, monitoring was mainly carried out through simulated artificial rainfall to reveal the distribution law of rainfall in soil water (SoW), plant water (PW), surface water (SW), and ground water (GW). They provide a reference for evaluating PW consumption and efficient utilization of water resources in karst desertification areas. In order to study the conversion rate of five water in rocky desertification area, in a study by Lu et al. [34], the researchers designed a runoff plot with a length of 5 m, width of 4 m, and height of 0.6 m to investigate the conversion rates of five water under different rock exposure rate, vegetation coverage, soil thickness, and slopes in the karst desertification area in northern Guangdong, China. By monitoring 57 instances of simulated rainfall (rainfall intensity 36–72 mm h^−1^), they observed the following results: SW accounted for 48.7–63.5 % of rainfall, vegetation interception accounting for 5.3.3–33.6 %, SoW content accounting for 5.2–22.6 %, and GW accounting for 5.6–9.8 %. Another field trial study of five-water conversion in karst areas showed that the conversion rates of precipitation to SW, PW, SoW, and GW were 3.5–6%, 1.5–3.5 %, 20–70 %, and 25–50 %, respectively [26]. The conversion rates of five water in these two karst areas were different. In the former, SW had the highest conversion rate, while in the latter, SoW had the highest conversion rate.

The difference between the above results are attributed to the scale researched in the two studies: The former obtained its data from simulated rainfall monitoring while the latter from basin scale monitoring. This study monitored the five-water conversion on the field scale by integrating the study of precipitation, soil water (SoW), plant water (PW), surface water (SW), and ground water (GW) in agroforestry systems, aiming to reveal the transformation mechanism of five water and the water consumption law of crops in the growing period, and thereby creatively proposes effective strategies of realizing efficient utilization of water resources in agroforestry. The conclusions provide an important reference for developing water-saving and value-added ecological industries, resisting karst drought, and preventing karst desertification in karst regions. They also add to the five-water conversion theory.

## 2 Materials and methods

### 2.1 Study areas

**Fig. 1:**
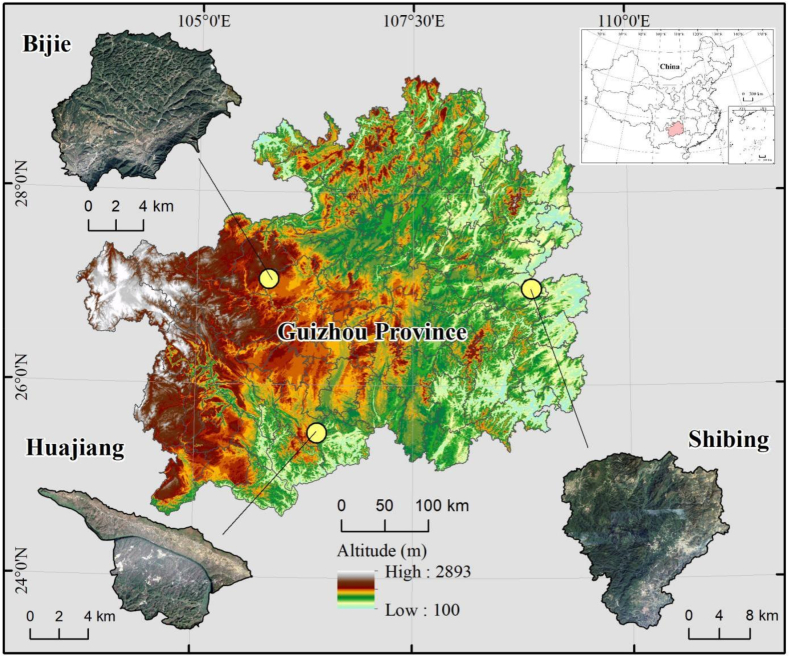
Location of the study area.

The Bijie Salaxi Research Area (Bijie) is located in the northwest of Guizhou Province (105°02′01″-105°08′09″E, 27°11′36″-27°16′51″N). It covers an area of 8627.19 hm^2^ (73.94 % is karst) and the altitude reaches 1509–2180 m. It has an average annual temperature of 12 °C and average annual precipitation of 984.4 mm. Mostly made of limestone, this region is typical of potential-mild karst desertification of karst plateau. The Guanling–Zhenfeng Huajiang Research Area (Huajiang) is in the southwest of Guizhou Province (105°36′30″-105°46′30″E, 25°39′13″-25°41′00″N). It has an area of 5161.65 hm^2^ (87.92 % is karst), is situated about 450–1450 m above sea level, and has an annual mean temperature of 18.4 °C and average rainfall of 1100 mm. Dominated by dolomitic limestone as well, it represents typical medium-intensity karst desertification in a karst plateau canyon. The Shibing Research Area (Shibing) is in the eastern part of Guizhou Province (108°01′36″-108°10′52″E, 27°13′56″-27°04′51″N), with an area of 28295 hm^2^, 89.11 % of which is karst. It is situated at 600–1250 m above sea level, and has an annual average temperature of 16 °C, with annual mean precipitation of 1220 mm. It is a region without-potential karst desertification potential in a dolomite plateau valley (Fig. 1).

### 2.2 Experimental design

#### 2.2.1 Data collection

At the 3 study sites, we delineated field runoff plots (3 m × 12 m) with a slope of 10°. The plots were selected based on varying vegetation cover, including grassland, agroforestry, and corn land. The distribution of runoff plots includes 9 in Bijie, 2 in Huajiang and 4 in Shibing research areas, respectively. They are numbered as Jb_1-9,_Jh_1-2,_Js_1-4_ (Table 1). Jb_2_ and Jb_3_ were set as control groups to compare the runoff yield of land use with no tillage and varying vegetation coverage; Jb_6_ and Jb_7_ were used to compare the characteristics of infiltration-excess and saturation-excess runoff under the same vegetation cover. Jb_7_ was built in a special way: a sunken V-shape area with a 120°angle was formed at the boundary between the sediment collecting trough and the soil in the runoff plot; yield above V-shape was infiltration-excess runoff, which overflowed out of the V and entered the collecting pool, generating saturation-excess runoff. Crops were planted in December 2018, with the exception of plot Jb_1_, which was planted in March 2019. From April to August in 2019, data were collected in these plots by monitoring surface runoff, soil evaporation, soil water (SoW) content, plant transpiration, and vegetable interception.

**Table 1:** Basic situation of runoff plots in the study areas.

Study area	Runoff plot number	Vegetation	Row space (m)	Vegetation coverage (%)	Soil type
Bijie	Jb_1_	Maize	0.6 × 0.6	70	Sandy yellow earth
	Jb_2_	Weeds	no	60	Sandy yellow earth
	Jb_3_	Weeds	no	70	Sandy yellow earth
	Jb_4_	Roxburgh rose + Plum	2 × 2	70	Sandy yellow earth
	Jb_5_	Maize + Perennial ryegrass	0.6 × 0.6	50	Sandy yellow-brown earth
	Jb_6_	Perennial ryegrass	Sowing	80	Yellow-brown earth
	Jb_7_	Perennial ryegrass	Sowing	80	Yellow-brown earth
	Jb_8_	Roxburgh rose + Perennial ryegrass	2 × 2	70	Yellow-brown earth
	Jb_9_	Mulberry + Orchardgrass	2 × 2	80	Yellow-brown earth
Huajiang	Jh_1_	Paper mulberry + Peanut	2 × 2	70	Yellow earth
	Jh_2_	Paper mulberry + *Euchresta japonica*	2 × 2	60	Yellow earth
Shibing	Js_1_	Pear + Perennial ryegrass	2 × 2	80	Brown earth
	Js_2_	Pear + Soybean	2 × 2	70	Brown earth
	Js_3_	Pear + *Radix pseudostellariae*	2 × 2	70	Yellow earth
	Js_4_	Pear	2 × 2	60	Brown earth

Surface runoff was obtained from a total of 13 runoff plots in the 3 study areas. During April–August 2019, measuring cylinders with a volume of 2000 ml were used to directly measure the yield of surface runoff in runoff ponds within 8 h after erosive rainfall events. In the same period, ring knife sampling was carried out once a month in the runoff plots of the 3 study areas in the 0–60 cm soil layer (divided into 3 layers: 0–20, 20–40, and 40–60 cm). Soil was collected from the upper, middle, and lower parts of the runoff plot according to an S shape. The collected soil was analyzed in the laboratory to obtain its physical properties of soil water (SoW) content, soil porosity, and soil field capacity.

**Fig. 2:**
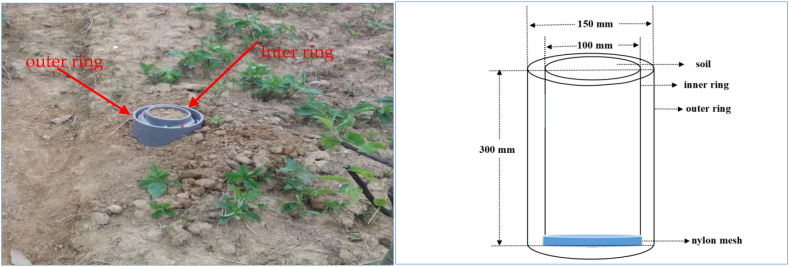
Self-made microlysimeter.

Self-made microlysimeters were used to monitor soil evaporation. On the days without rain in the mid-April to mid-August 2019, the microlysimeters were installed in each runoff plot, in the parts with and without vegetation cover. Consisting of inner and outer rings, the microlysimeters were made of polyvinyl chloride (PVC). The inner ring was 300 mm long and 100 mm in diameter (cross-sectional area: 78.5 mm^2^), with 3 mm wall thickness, while the outer ring was 300 mm long and 150 mm in diameter, 3 mm wall thickness (Fig. 2). At 8:00 on the first day of each monitoring period, the inner ring was hammered into the soil, forming a column of soil within it. Then, with a hoe, we dug out the inner ring along with the soil column, being very careful to protect the soil column from damage, and cleaned up the soil on the outer wall. After that, we used nylon netting to wrap the bottom of the microlysimeter to prevent soil from leaking. Third, we weighed the inner ring with the soil column on an electronic balance brought to the site. The balance measures up to 5 kg and is accurate to 0.01 g. Fourth, we installed the outer ring vertically into the pit where the column was excavated so that the upper part was flush with the ground. We also put scrap newspaper inside the outer ring to prevent soil from sticking to the bottom of the inner ring. Lastly, we placed the inner ring inside the outer ring. At the same time the next morning, we took out the inner ring and weighed it again. The difference between the two weights on the two days was the 24 h soil evaporation (g). We measured the average daily soil evaporation for 3 consecutive days in each runoff plot every month.

We conducted real-time monitoring of precipitation and temperature at the three study sites using a small meteorological station (David Vantage Pro) with a recording frequency of once every 10 min. The collected data were calculated with the Koichiro–Takahashi formula (Eq. (1)) [[35], [36], [37]] to obtain land evapotranspiration (LE). LE is the process of land and surface water (SW) evaporation. It is a key process in the climatic and biogeochemical cycles of terrestrial ecosystems and is an essential part of the hydrological cycle, energy balance, and carbon cycle [[38], [39], [40]].

(1) $$E=\frac{3100P}{3100+1.8P^{2}\;\exp\;\left(-\frac{34.4t}{235+t}\right)}$$

here, *E* is monthly LE (mm), *P* is total monthly precipitation (mm), and *t* represents monthly average temperature (°C). With this formula, we can calculate available precipitation *F* (mm) (Eq. (2)), evapotranspiration coefficient *α* (Eq. (3)), and available precipitation coefficient *β* (Eq. (4)):

(2) F=P−E

(3) α=E/P

(4) β=(P−E)/P

Rainfall is the sum of available precipitation and LE. Available precipitation provides water for crop growth and can be transformed into surface water (SW), ground water (GW), soil water (SoW), and plant water (PW). It reflects the abundance of water resources in a region and is a form of water resource storage. LE occurs mainly in the process of liquid water converting into vapor, including soil evaporation, plant transpiration, and vegetation interception, and it can be seen as a form of water resource conversion.

Pruning and weighing were employed to assess the crop transpiration rates and vegetation interception of agroforestry [41,42]. Measurements were conducted in the research plots from April to August 2019, 3 consecutive days every month. First, we placed a wind-proof electronic balance (with precision of 0.001) in a relatively flat place near the crops to be monitored in the field. We then cut off standard branches and immediately put them on the balance to weigh. Here, standard branches are branches or leaves at the top of a tree or crop, all with similar diameter, branch length, and leaf weight. Third, the weighed branches were returned to their respective places and weighed again 5 min later. The difference between the two weights was the transpiration rate and amount (Eq. (5)). The branches were weighed once every 2 h from 8:00 in the morning to 6:00 in the evening, for a total of 6 times a day. After the last weighing, we immediately immersed the branches and leaves in water for 5 min and then took them out. About 3 min later, when the branches and leaves stopped dripping, they were put into a plastic bag for weighing. Removing the weight of the plastic bag, we calculated the difference in weight before and after immersion, and obtained the weight of intercepted water (plant water (PW) capacity) (Eq. (6)) [43]:

(5) $$Et=\frac{m_{0}-m_{1}}{\left(m_{0}-m_{z}\right)\times 5}\times 60$$

where *Et* is the transpiration rate (g g^−1^ h^−1^), *m*_*0*_ is the initial weight of branches and leaves (g), *m*_*1*_ is the final weight of branches and leaves (g), and *m*_*z*_ is the weight of branches (g).

(6) Iv=Pb×Rw/S×n

Here, *Iv* is the amount of vegetation interception (mm), *Pb* is crop biomass (g), *Rw* is plant water (PW) holding rate, *S* is the sample area (m^2^), and *n* is the amount of rainfall.

We monitored crop biomass and dry matter to calculate Water use efciency (WUE). Biomass is constantly changing, so it was obtained by the harvest method [[44], [45], [46]]. In order to ensure that we could carry out continuous positioning monitoring in the future, we selected crops that had the same stand, species, average plant height, stand age, density, and planting method from adjacent plots (4 m × 5 m). These plots were 5 m apart from each other and had similar altitude, slope soil type, and agronomic measures applied. The aboveground and underground parts of all crops were harvested and the fresh weight was obtained. They were then taken back to the laboratory for dry weight analysis. The last harvested crops were measured to obtain the fresh weight and dry weight of the biomass, so as to assess the WUE (Eq. (7)) [47].

(7) WUE=M/ET

where WUE is water use efficiency (kg m^−3^), M is biological dry matter content (kg), and ET is evapotranspiration (mm) (the sum of soil and crop evaporation).

Ground water (GW) was calculated by the water balance equation (Eq. (8)):

(8) Wg=P−Wi−Wr−Ws

where *Wg* is GW (mm), *P* is rainfall (mm), *Wi* is the amount of vegetation interception (mm), *Wr* is SW (mm), and *Ws* is SoW (mm).

#### 2.2.2 Data analysis

WPS Office 2022 was used for data analysis and mapping. IBM SPSS Statistics 23 was used for correlation analysis, 2-tailed testing and analysis of variance (ANOVA). The correlation coefficient is denoted by the letter r (0 ≤ |r | ≤ 1). A perfect correlation occurs when the absolute value of r is equal to 1. The closer the absolute value of r is to 1, the higher the correlation; conversely, the closer it is to 0, the lower the correlation. It is generally suggested that the correlation is high when the absolute value of r is greater than or equal to 0.7 but less than 1 (0.7 ≤ |r | ＜ 1), moderate if it is greater than or equal to 0.4 but less than 0.7 (0.4 ≤ |r | ＜ 0.7), and low when it is greater than or equal to 0.1 but less than 0.2 (0.1 ≤ |r | ＜ 0.2). P represents the significance value of the two-tailed test. Significant correlation exists when p is smaller than or equal to 0.01 (p ≤ 0.01). ANOVA was applied to analyze differences between data groups.

## 3 Results

### 3.1 Transformation characteristics of precipitation

Table 2 illustrates the storage and transformation of precipitation in the three study areas in 2019. The total rainfall during this year in Bijie was 1425.20 mm, reaching a peak in July and the lowest level in December. In Huajiang it was 1088.4 mm, with the most in September and the least in February. In Shibing, it was 1341.5 mm in total, with a similar distribution to the other two, the maximum in summer and minimum in winter. The available precipitation in Bijie reached a total of 810.52 mm (β = 0.57) that year, peaking in July and reaching the lowest level in December, with land evapotranspiration equal to 614.68 mm (α = 0.43). Huajiang received a total of 409.93 mm (β = 0.38) available precipitation, with the most in September and the least in February, and LE was 678.47 mm (α = 0.62). Shibing was between them, with a total of 701.91 mm (β = 0.52) and LE of 639.59 mm (α = 0.48) (Table 2). It is observed from the data that less water was stored and more water resources were converted in the Huajiang research area, and in Bijie the reverse occurred.

**Table 2:** Storage (or transformation) characteristics of precipitation water in 2019 in three study areas.

Month	Precipitation (mm)			Available precipitation (mm)			LE (mm)		
	Bijie	Huajiang	Shibing	Bijie	Huajiang	Shibing	Bijie	Huajiang	Shibing
Jan.	31.00	17.00	42.00	6.94	0.51	16.93	24.06	16.49	25.07
Feb.	45.00	10.40	37.00	8.51	0.08	12.84	36.49	10.32	24.16
Mar.	64.00	12.60	64.00	18.89	0.09	23.32	45.11	12.51	40.68
Apr.	88.00	33.50	112.80	28.41	0.66	52.24	59.59	32.84	60.56
May	107.00	31.20	171.60	39.39	0.55	107.56	67.61	30.65	64.04
Jun.	196.60	253.00	243.80	125.27	127.93	157.99	71.33	125.07	85.81
Jul.	304.00	156.60	216.60	243.73	44.13	119.01	60.27	112.47	97.59
Aug.	286.00	75.60	258.20	200.67	5.51	154.67	85.33	70.09	103.53
Sep.	151.00	296.40	31.20	78.25	190.62	1.00	72.75	105.78	30.20
Oct.	92.00	101.00	111.80	37.35	20.23	50.61	54.65	80.77	61.19
Nov.	48.00	57.60	21.00	22.46	12.17	0.83	25.54	45.43	20.17
Dec.	12.60	43.50	31.50	0.66	7.45	4.91	11.94	36.05	26.59
Total	1425.2	1088.40	1341.50	810.53	409.93	701.91	614.67	678.47	639.59

Correlation analysis was performed to examine the relationships between precipitation, LE, and available precipitation in the three research areas, and the results show significantly positive relationships between precipitation and available precipitation (r = 0.970, P ＜ 0.01), precipitation and LE (r = 0.852, P ＜ 0.01), and available precipitation and LE (r = 0.700, P ＜ 0.01).

### *3.2* *Storage and transformation of* surface water (SW) *and efficient use of agroforestry*

In the 2019 crop growing season, the total erosive rainfall in Bijie, Huajiang, and Shibing was 776, 158.80, and 729.80 mm, respectively (shown in Table 3), and different runoff yields were observed among the plots in the three study areas. In Jb_9,_ orchardgrass (*Dactylis glomerata* L.) had been growing for 5 years and covered almost all of the surface soil, with thin roots seen everywhere in the 10 cm soil layer. Thus, rainfall had permeated considerably, leading to less surface runoff. Considering the efficient utilization of precipitation resources through agroforestry, this indicates that this mode is the most optimal in the three research areas. In Jb_4_, runoff was generated in each of 15 erosive rainfalls, reaching a depth of 20.28 mm, with a coefficient of 0.03 and a runoff modulus of 20,277.78 m^3^ km^−2^ a^−1^, second only to the runoff produced in Jb_7_, which had been treated with engineering measures. The surface runoff depth of Jb_7_ was 15 mm, the runoff coefficient was 0.02, and the runoff modulus was 15000 m^3^ km^−2^ a^−1^. This provides evidence that engineering measures have an immediate effect on soil and water conservation. Jb_8_ yielded runoff that was only less than Jb_5_, as perennial ryegrass (*Lolium perenne* L.) was planted in its first year, the roots of which had not created a network-fixation effect. As a result, small gullies appeared due to erosion by rainwater, resulting in soil crust and then reduced infiltration. Jb_2_ and Jb_3_, both weedy runoff plots, were almost abandoned land, where a negative relationship was found between surface runoff and vegetation coverage: the larger the coverage, the smaller the runoff. Jb_5_ produced more surface runoff than the other agroforestry modes (Jb_4_, Jb_8_, and Jb_9_), and even more than Jb_1_, since it was planted later, resulting in poorer growth and thinner coverage. In summary, regarding the efficiency of water resource utilization in different agroforestry modes, it was found in the Bijie study area that Jb_9_ was more efficient than Jb_4_, followed by Jb_8_.

**Table 3:** Characteristics of different runoff plots in the study areas.

Study area	Runoff plots	Erosive rainfall (mm)	Runoff yield (m3)	Runoff depth (mm)	Runoff coefficient	Runoff modulus (m3 km^-^2 a^−1^)
Biejie	Jb1	776.00	1.51	41.94	0.05	41,944.44
	Jb2	776.00	3.24	90.00	0.12	90,000.00
	Jb3	776.00	2.80	77.78	0.10	77,777.78
	Jb_4_	776.00	0.73	20.28	0.03	20,277.78
	Jb_5_	776.00	4.49	124.72	0.16	124,722.22
	Jb_6_	776.00	1.96	54.44	0.07	54,444.44
	Jb_7_	776.00	0.54	15.00	0.02	15,000.00
	Jb_8_	776.00	3.33	92.50	0.12	92,500.00
	Jb_9_	776.00	0.02	1.40	0.003	1400.00
Huajiang	Jh_1_	158.80	0.63	17.50	0.11	17,500.00
	Jh_2_	158.80	0.45	12.50	0.08	12,500.00
Shibing	Js_1_	729.80	3.46	96.11	0.13	96,111.11
	Js_2_	729.80	3.95	109.72	0.15	109,722.22
	Js3	729.80	4.12	114.44	0.16	114,444.44
	Js4	729.80	4.52	125.56	0.17	125,555.56

In the Huajiang research area, Jh_1_ had a better effect on curbing surface runoff than Jh_2_, while in Shibing, Js_1_ was the most effective, followed by Js_2_, Js_3_, and Js_4_. Among them, a larger runoff modulus was seen for Js_3_, because its water permeability was reduced by ridge farming along the slope. The soil here was mostly hardened and compacted by people constantly moving between the rows of *Radix pseudostellariae*. When comparing all modes in the three research regions, it was shown that forest + grass had the best performance not only in reducing surface runoff and increasing soil infiltration, but in efficiently utilizing water resources by agroforestry. Overall, agroforestry does help to reduce surface runoff and increase soil infiltration.

### *3.3* *Storage and transformation of* soil water (SoW) *and efficient utilization of agroforestry*

**Fig. 3:**
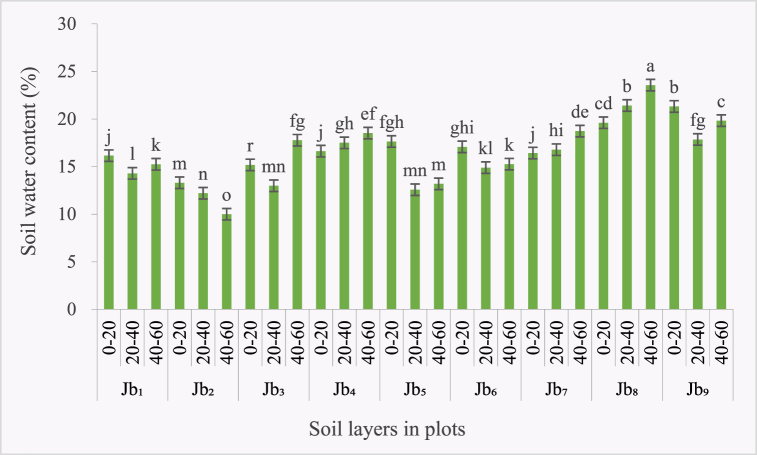
Vertical distribution of soil water (SoW) content in Bijie study area (In the figure, Jb_1-9_ represents the 9 runoff plots in Bijie.).

The average soil water (SoW) content was 16.51 % in the Bijie research area. Between plots in this region, the lowest content (10 %) was found in the 60 % covered weed plot (Jb_2_) in the 40–60 cm soil layer. The highest content (23.56 %) was measured in in 40–60 cm soil layer of Jb_8_ (Fig. 3). Vertically, the highest SoW content was found in the 0–20 cm soil layer (17.03 %), followed by the 40–60 cm layer (16.61 %) and the 20–40 cm layer (15.61 %). Between modes, the SoW content was higher in the agroforestry area (19.58 %) than the non-agroforestry area (14.98 %). In Huajiang, the average SoW content was 17.83 %, similar to that of Bijie. The vertical distribution showed that the upper layer (20.06 %) contained higher SoW content than the bottom layer (17.32 %), and the middle layer (16.10 %) had the least. This is another similarity between the two study areas: the lowest content of SoW was found in the middle layer of soil. The reason may be that the roots of crops are mainly distributed in the middle layer, where they absorb the water.

**Fig. 4:**
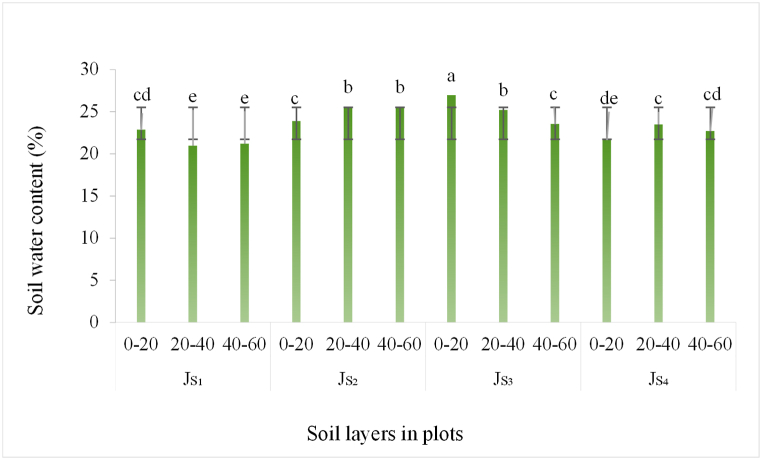
Vertical distribution of soil water (SoW) content in Shibing study area (In the figure, Js_1-4_ represents the 4 runoff plots in Huajiang.).

The research plots in Shibing contained 23.61 % average soil water (SoW) content, with the lowest (20.96 %) in Js_1_ in the 20–40 cm soil layer and the highest (26.95 %) in Js_3_ in the 0–20 cm layer (Fig. 4). Vertically, the average SoW content decreased with depth, with 23.82 % at 0–20 cm, 23.79 % at 20–40 cm, and 23.24 % at 40–60 cm, gradually decreasing from the upper layer to the bottom layer, but not by much. This may suggest that there is little effect of root water absorption on SoW content if there is abundant SoW. Horizontally, the content was higher in agroforestry (23.95 %) than non-agroforestry (22.73 %), confirming the significant benefit of agroforestry systems on SoW retention.

Analysis of variance showed that there was no significant difference in the vertical distribution of soil water content among the three study areas (p > 0.05). The monthly average soil water contents of the three areas were different overall from April to August in 2019, with May, July and August showing no significant difference (17.20 ± 3.85 c, 18.27 ± 3.84 b, 20.30 ± 4.66 a, 18.92 ± 4.23 b, 18.22 ± 4.05 b). Between groups, soil water content showed significant diference among Shibing (23.61 ± 3.26 a), Huajiang (17.83 ± 2.58 b), and Bijie (16.94 ± 3.26 c).

Examining soil evaporation of all crops including monoculture, Shibing and Bijie had similar distribution. The highest was seen in July in Js_2_ (157.65 ± 0.86 mm) and August in Jb_2_ (149.10 ± 0.69 mm), while the lowest was in April in Js_3_ (15.09 ± 0.37 mm) and Jb_4_ (6.06 ± 0.78 mm). In both regions, there was less soil evaporation from April to June than from July to August, and perennial ryegrass agroforestry had a better effect on inhibiting soil evaporation than other modes. In contrast to Bijie and Shibing, Huajiang saw higher evaporation in April to May than June to August, as it rained less during this period. The average soil evaporation of the three study areas was as follows: the lowest was in April (28.99 ± 31.02 mm) and the highest was in August (110.70 ± 25.51 mm), with median values in May (45.44 ± 36.62 mm), June (55.59 ± 29.73 mm), and July (106.27 ± 36.22 mm) (Table 4). The average evaporation amount was 346.98 ± 71.39 mm in the three areas. Specifically, from April to August, Bijie study plots showed 320 ± 68.60 mm average evaporation, Huajiang was up to 389.42 ± 29.55 mm, and Shibing reached 386.26 ± 66.43 mm. After excluding monoculture, the average soil evaporation of agroforestry was as follows: the highest was in Shibing (395 ± 51.66 mm), followed by Huajiang (389 ± 29.55 mm) and Bijie (288 ± 63.08 mm). These results correspond to soil water (SoW) content, indicating that a sufficient water supply is a necessary condition for evaporation. In addition, it was also found that agroforestry had a better effect on controlling soil evaporation than monoculture. For instance, in Bijie, a lower average evaporation rate was found in agroforestry systems (2.01 mm d^−1^) than other treatments (2.37 mm d^−1^).

**Table 4:** Soil evaporation (mm) in different planting modes in the study area from April to August.

Modes	Apr.	May	Jun.	Jul.	Aug.	Total
Jb₁	20.45 ± 0.52	74.54 ± 0.49	34.11 ± 0.80	124.02 ± 1.16	115.92 ± 0.23	369.05 ± 0.65
Jb₂	20.87 ± 0.16	65.70 ± 0.67	55.05 ± 0.49	141.07 ± 1.48	149.10 ± 0.69	431.80 ± 2.22
Jb₃	15.40 ± 0.22	65.79 ± 1.08	58.70 ± 1.02	134.08 ± 1.17	89.74 ± 1.38	363.71 ± 2.49
Jb₄	6.06 ± 0.78	32.28 ± 0.37	40.08 ± 0.98	126.15 ± 0.28	123.52 ± 0.56	328.09 ± 0.41
Jb₅	25.94 ± 0.07	21.69 ± 0.88	71.24 ± 0.33	128.47 ± 0.77	78.40 ± 0.75	325.74 ± 0.72
Jb₆	16.45 ± 0.06	19.66 ± 0.95	41.94 ± 1.01	90.19 ± 0.22	98.00 ± 0.81	266.25 ± 2.46
Jb₇	15.36 ± 0.46	18.47 ± 0.55	23.96 ± 1.14	94.83 ± 1.22	105.57 ± 0.42	258.20 ± 2.50
Jb₈	17.57 ± 0.34	14.49 ± 0.08	20.29 ± 0.39	55.68 ± 1.36	84.01 ± 0.21	192.04 ± 1.10
Jb₉	15.18 ± 0.09	57.32 ± 0.87	23.69 ± 0.54	114.29 ± 0.46	135.51 ± 0.56	345.99 ± 0.81
Jh₁	102.47 ± 1.23	107.43 ± 0.67	34.51 ± 0.30	37.05 ± 0.18	81.06 ± 1.03	362.52 ± 1.91
Jh₂	112.10 ± 0.99	135.78 ± 2.69	47.99 ± 0.55	37.44 ± 0.53	83.02 ± 0.05	416.33 ± 2.73
Js₁	16.55 ± 0.76	16.80 ± 0.45	71.14 ± 1.98	99.77 ± 1.05	99.42 ± 0.71	303.67 ± 2.59
Js₂	17.30 ± 0.24	17.91 ± 0.30	130.25 ± 1.13	157.65 ± 0.86	154.54 ± 0.49	477.66 ± 2.70
Js₃	15.09 ± 0.37	16.48 ± 0.15	83.26 ± 0.84	141.37 ± 1.13	147.89 ± 0.82	404.10 ± 1.78
Js₄	18.06 ± 0.55	17.24 ± 0.22	97.59 ± 0.99	111.97 ± 0.53	114.76 ± 0.83	359.62 ± 1.43
Average	28.99 ± 31.02	45.44 ± 36.62	55.59 ± 29.73	106.27 ± 36.22	110.70 ± 25.51	346.98 ± 70.52

### *3.4* *Storage and transformation of* plant water (PW) *and efficient utilization of agroforestry*

**Fig. 5:**
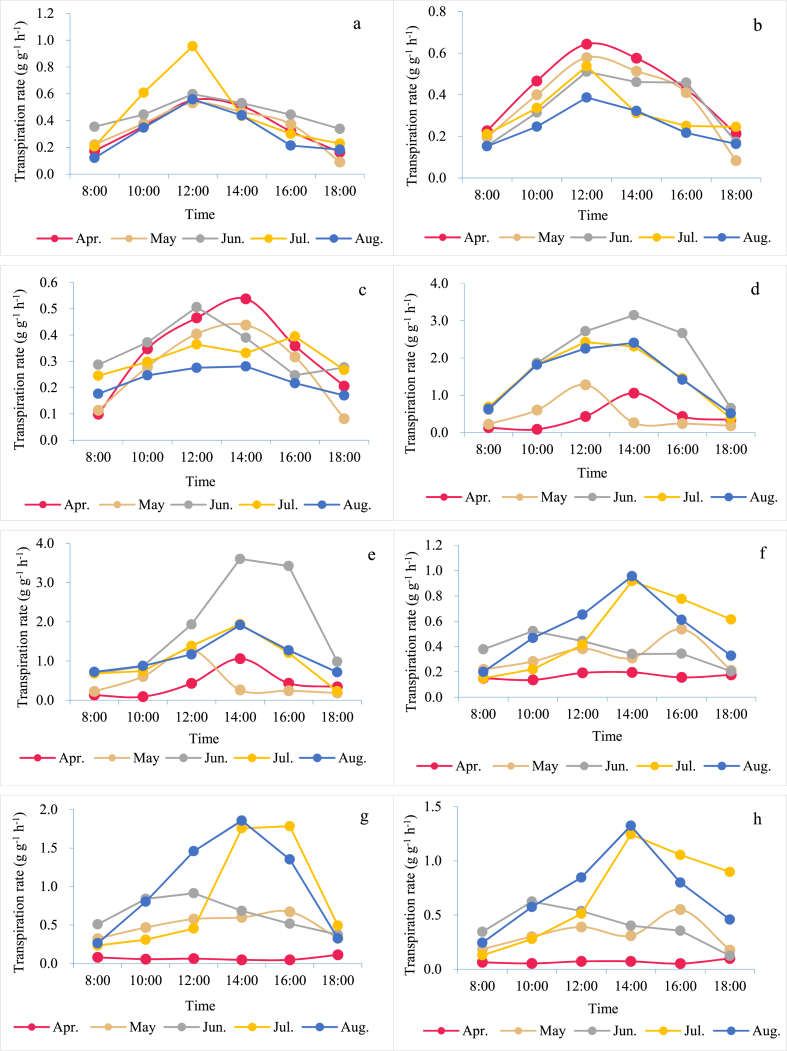
Transpiration rate of agroforestry systems: (a) Jb_4_, (b) Jb_8_, (c) Jb_9_, (d) Jh_1_, (e) Jh_2_, (f) Js_1_, (g) Js_2_, (h) Js_3_.

The transpiration rate in the agroforestry system (Fig. 5) was obtained by calculating the rates of its two crops with the weighted mean method. It was found that the transpiration rate of agroforestry was higher than that of one crop, but lower than the average of both crops. Of the three study areas, the transpiration rate was the highest in Huajiang (1.077 g g^−1^ h^−1^), followed by Shibing (0.476 g g^−1^ h^−1^), and the lowest was in Bijie (0.300 g g^−1^ h^−1^). Among modes, the maximum was found in Jh_1_ (1.168 g g^−1^ h^−1^) and the minimum in Jb_9_ (0.300 g g^−1^ h^−1^). This may be attributed to the more intense solar radiation and higher temperature in Huajiang, which results in fast transpiration. By comparison, the transpiration rate of agroforestry was lower than that of monoculture, and agroforestry does contribute to WUE by reducing transpiration.

Regionally, in Bijie, transpiration in the agroforestry system was the lowest in Jb_4_ (83.00 ± 1.34 mm; Table 5), as the two crops carry the least biomass. In terms of total transpiration, the highest transpiration was in Jb_8_ (resulting from its massive biomass), followed by Jb_9_ and Jb_4_. In Huajiang, Jh_1_ showed the highest transpiration. The paper mulberry biomass in the two runoff plots was similar. This discrepancy between Jh_1_ and Jh_2_ was probably caused by peanut and sub-prostrate sophora, which have different transpiration rates and biomass. The transpiration in the former was more than 4 times higher than in the latter, and it had larger biomass as well. In Shibing, the most intense transpiration (604.60 ± 0.78 mm) was in Js_1_ because, similar to Bijie, perennial ryegrass grows rapidly and has abundant biomass as well as higher transpiration. In Js_1_–Js_4_, pear trees received the same treatment and had the same biomass, and their transpiration rates in monoculture were faster than those in agroforestry. Nevertheless, the amount of transpiration was found to be less in monoculture than the agroforestry system due to the latter's larger biomass. Among the three research regions, transpiration was the highest in Shibing, followed by Bijie and Huajiang, significantly correlating with biomass (r = 0.883, P < 0.01). It is interesting that WUE was the opposite: in Huajiang (0.74 kg t^−1^) it was the most effective, while in Shibing (0.60 kg t^−1^) it was the least, and Bijie (0.70 kg t^−1^) was in the middle, which indicates the role of drought stress in improving crop WUE [48]. In Huajiang, 7 month drought slowed down the growth of paper mulberry, peanut, and sub-prostrate sophora to varying degrees. With abundant rainfall in the following months, these crops sprung up rapidly, giving rise to the highest WUE.

**Table 5:** Crop transpiration (mm) in the study areas.

Modes	Apr.	May	Jun.	Jul.	Aug.	Total (mm)
Jb1	21.31 ± 1.26	24.32 ± 0.68	30.65 ± 0.83	80.81 ± 1.46	77.30 ± 0.25	234.38 ± 1.67
Jb4	15.14 ± 0.42	16.61 ± 0.55	16.03 ± 0.22	17.98 ± 0.41	17.25 ± 0.99	83.00 ± 1.34
Jb5	42.59 ± 0.53	50.07 ± 0.42	48.66 ± 0.88	54.71 ± 0.41	59.29 ± 0.83	255.31 ± 1.28
Jb6	67.12 ± 0.25	76.46 ± 0.67	73.70 ± 1.02	80.96 ± 0.44	78.80 ± 0.93	377.05 ± 1.61
Jb7	73.04 ± 0.51	76.06 ± 0.73	75.99 ± 1.40	79.74 ± 0.67	75.96 ± 1.30	380.78 ± 2.98
Jb8	74.71 ± 1.07	78.13 ± 0.44	78.24 ± 0.82	82.50 ± 0.86	78.96 ± 0.66	392.54 ± 2.02
Jb9	43.91 ± 0.80	53.11 ± 0.73	53.12 ± 0.21	70.92 ± 0.62	79.98 ± 0.85	301.03 ± 1.67
Jh1	19.51 ± 0.34	20.31 ± 0.11	47.33 ± 0.83	50.27 ± 1.10	56.29 ± 0.89	193.70 ± 2.27
Jh2	19.51 ± 1.12	20.31 ± 0.40	20.83 ± 0.53	20.90 ± 0.85	27.59 ± 0.61	109.13 ± 0.63
Js1	108.06 ± 0.76	125.38 ± 0.43	112.75 ± 0.62	138.82 ± 1.06	119.59 ± 1.76	604.60 ± 0.78
Js2	15.82 ± 0.36	100.59 ± 0.52	104.13 ± 1.13	129.19 ± 1.00	119.49 ± 1.05	469.23 ± 1.79
Js3	31.57 ± 0.61	40.38 ± 0.93	37.59 ± 0.55	30.63 ± 1.74	24.38 ± 0.55	164.54 ± 3.79
Js4	15.82 ± 0.65	22.82 ± 1.02	22.79 ± 0.60	30.63 ± 0.93	24.38 ± 0.81	116.43 ± 0.66

As shown in Table 6, the vegetation interception varied widely among planting modes in the whole crop growing season but was positively correlated with biomass and rainfall. Among the modes, Js_1_ in Huajiang retained rainfall the most effectively, benefiting from the highest biomass carried by the two crops, pear tree and ryegrass, as well as the high water holding capacity of ryegrass (0.93). Another reason is that Shibing received the most rainfall. Jb_6_–Jb_8_ in Bijie had an outstanding effect on intercepting rainfall, on account of one crop, ryegrass, which proved to have strong water-holding capacity and a large quantity of biomass. Another crop, maize, also showed an excellent capacity to catch water (0.91) and contained tremendous biomass, bringing about a higher rate of vegetation interception, whether in monoculture or agroforestry interbred with ryegrass. Jb_4_ was the opposite, with the least biomass and weak water retention ability, thus leading to the lowest interception rate. In Huajiang, a lower interception rate was found in all modes because of the small biomass content of Jh_1_ and Jh_2_. Comparing the average interception values of three regions, Shibing was excellent at 42.78 ± 33.44 mm and Huajiang was the lowest at 10.64 ± 3.35 mm, with Bijie in the middle at 34.43 ± 16.77 mm, revealing a coupling relationship with the regional distribution of biomass and rainfall. The implication here is that in order to reduce vegetation interception and increase the available precipitation, pruning and other agronomic techniques are highly recommended to improve the efficiency of water resource utilization in agroforestry.

**Table 6:** Vegetation interception (mm) in the study areas.

Modes	Apr.	May	Jun.	Jul.	Aug.	Total
Jb_1_	3.36 ± 0.13	1.97 ± 0.11	3.34 ± 0.46	11.47 ± 0.51	8.11 ± 0.29	28.24 ± 0.77
Jb_4_	3.17 ± 0.36	1.80 ± 0.18	2.32 ± 0.20	3.39 ± 0.09	2.44 ± 0.21	13.11 ± 0.92
Jb_5_	7.35 ± 0.53	4.44 ± 0.21	5.78 ± 0.58	8.45 ± 0.42	6.78 ± 0.15	32.80 ± 1.60
Jb_6_	11.86 ± 0.03	6.96 ± 0.23	9.00 ± 0.93	12.89 ± 0.37	9.28 ± 0.27	49.99 ± 1.28
Jb_7_	12.90 ± 0.78	6.92 ± 0.45	9.28 ± 0.26	12.70 ± 0.57	8.95 ± 0.42	50.75 ± 0.96
Jb_8_	13.74 ± 0.62	7.39 ± 0.58	9.89 ± 0.78	13.59 ± 0.64	9.57 ± 0.17	54.19 ± 1.07
Jb_9_	0.75 ± 0.03	1.31 ± 0.04	2.36 ± 0.19	4.10 ± 0.57	3.43 ± 0.22	11.96 ± 0.18
Jh_1_	0.71 ± 0.09	1.38 ± 0.09	4.68 ± 0.27	3.87 ± 0.30	3.06 ± 0.37	13.69 ± 0.47
Jh_2_	0.71 ± 0.15	1.38 ± 0.14	2.20 ± 0.12	1.72 ± 0.06	1.6060 ± 0.23	7.60 ± 0.28
Js_1_	16.91 ± 0.92	27.25 ± 1.37	22.54 ± 1.51	10.95 ± 1.14	20.70 ± 0.89	98.35 ± 0.35
Js_2_	1.70 ± 0.02	9.01 ± 0.92	8.58 ± 0.52	4.30 ± 0.07	8.42 ± 0.69	32.01 ± 1.21
Js_3_	5.59 ± 0.53	9.55 ± 0.22	7.96 ± 0.59	1.70 ± 0.59	2.96 ± 0.21	27.76 ± 1.03
Js_4_	1.70 ± 0.12	3.44 ± 0.37	3.19 ± 0.31	1.70 ± 0.48	2.96 ± 0.33	12.99 ± 0.62

### *3.5* *Storage and transformation of* ground water (GW) *and efficient utilization of agroforestry*

Rainfed agriculture covers most of the karst areas. In the dry season when the drought stress is intense, ground water (GW) transforms into soil water (SoW) to feed the crops. During the actual process, fissure water, like subterranean water, rises through vaporization and then condenses into liquid water in the soil layer to replenish SoW. Hence, deep-rooted crops can directly take in GW (fissure water). GW levels in this study were obtained with the water balance equation, based on monitoring and calculating precipitation, surface runoff, SoW content and evaporation, and plant water (PW) in the three study areas. The results prove that the storage of GW ultimately serves the growth of crops, and large GW stock means highly efficient utilization of water resources by crops.

**Fig. 6:**
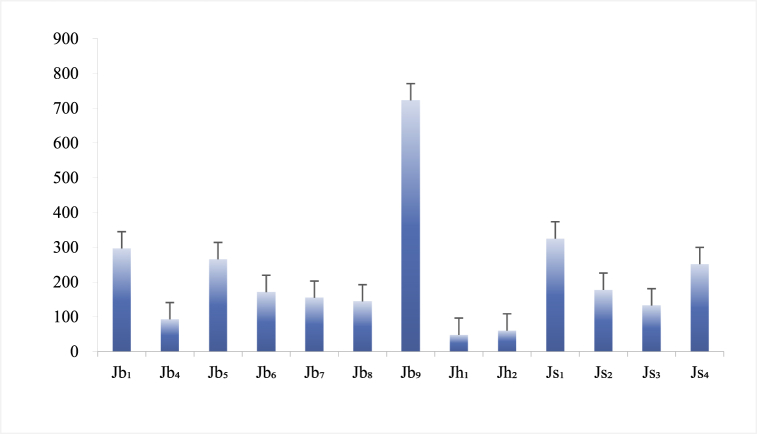
Ground water (GW) distribution in the study areas (In this figure, Jb_1-9_ refers to the 9 runoff plots in Bijie, Jh_1-2_ is the 2 runoff plots in Huajiang, and Js_1-4_ represents the 4 runoff plots in Shibing.).

Among the agroforestry systems in the three study areas (Fig. 6), rainfall rarely converted into GW in Jh_1_ and Jh_2_, for the critical reason that from April to August it rained the least in Huajiang during the crop growing season. Only 48.02 and 60.22 mm of rainfall converted into GW in Jh_1_ and Jh_2_, and the conversion coefficients were merely 0.09 and 0.11. On the contrary, the highest conversion coefficient (0.30) was found in Jb_9_, where there was little surface runoff and the majority of rainfall infiltrated through the soil to form GW. The second highest conversion rate (0.32) was found in Js_1_.

As for the ground water (GW) in Bijie, the most abundant storage was in Jb_9_, followed by Jb_8_, and the least in Jb_4_. in Huajiang, Jh_2_ had larger GW reserves than Jh_1_. In Shibing, there was more in Js_1_ than Js_2_ and Js_3_. GW conversion had a negative correlation with surface runoff and soil evaporation. Agroforestry, by lowering surface runoff and inhibiting soil evaporation, promotes subterranean water conversion, thereby realizing the efficient utilization of water resources.

### 3.6 Five-water storage and transformation through highly efficient utilization of agroforestry

Rainfall, as a total water resource, transforms into surface water (SW), plant water (PW), ground water (GW), and soil water (SoW) in the water circulation process. As shown in Table 7, the conversion rate of five water in Bijie, Huajiang, and Shibing, respectively, was as follows: SW was 0.14–12.71 %, 2.27–3.18 %, and 9.58–12.52 %; PW was 9.79–45.91 %, 21.23–37.71 %, and 12.90–70.08 %; GW was 9.43–73.58 %, 8.73–10.95 %, and 13.21–32.40 %; and SoW was 40.72–70.25 %, 75.01–82.58 %, and 59.98–76.70 %.

**Table 7:** Conversion rate of five water.

Modes	Rainfall (mm)	SW (mm)	SWCR (%)	PW (mm)	PWCR (%)	GW (mm)	GWCR (%)	SoW (mm)	SoWCR (%)
Jb₁	981.60	41.94	4.27	262.62	26.75	296.45	30.20	689.60	70.25
Jb₄	981.60	20.28	2.07	96.11	9.79	92.58	9.43	599.51	61.07
Jb₅	981.60	124.72	12.71	288.12	29.35	265.57	27.06	677.17	68.99
Jb₆	981.60	54.44	5.55	427.04	43.50	171.06	17.43	622.19	63.39
Jb₇	981.60	15.00	1.53	431.52	43.96	154.48	15.74	579.72	59.06
Jb₈	981.60	92.50	9.42	446.73	45.51	144.00	14.67	469.04	47.78
Jb₉	981.60	1.40	0.14	312.99	31.89	722.24	73.58	399.75	40.72
Jh₁	549.90	17.50	3.18	207.39	37.71	48.02	8.73	412.50	75.01
Jh₂	549.90	12.50	2.27	116.73	21.23	60.22	10.95	454.11	82.58
Js₁	1003.00	125.56	12.52	702.95	70.08	324.94	32.40	605.73	60.39
Js₂	1003.00	109.72	10.94	501.24	49.97	177.40	17.69	769.26	76.70
Js₃	1003.00	96.11	9.58	192.31	19.17	132.52	13.21	601.58	59.98
Js₄	1003.00	114.44	11.41	129.42	12.90	251.43	25.07	627.19	62.53

Note: SWCR is surface water conversion rate. PWCR is plant water conversion rate. GWCR is groundwater conversion rate. SoWCR is soil water conversion rate.

Obviously, the conversion of surface water (SW) and plant water (PW) was the highest in Shibing and the lowest in Huajiang. ground water (GW) conversion was the highest in Bijie and the lowest in Huajiang, and soil water (SoW) conversion was the highest in Huajiang and the lowest in Bijie. In Huajiang, agroforestry promoted a reduction in surface runoff; meanwhile, drought stress in this area lowered the rate of rainfall converting into plant and subterranean water, increased SoW conversion, and improved the efficiency of water use. As for the Water use efciency (WUE) of agroforestry, it manifested as follows: Huajiang (0.73 kg t^−1^) > Bijie (0.64 kg t^−1^) > Shibing (0.58 kg t^−1^). The average conversion rate of the three areas was 0.14–12.71 % for SW, 40.72–82.58 % for SoW, 8.73–30.20 % for GW, and 9.79–70.08 % for PW. Among them, SoW had the highest average conversion rate, which meets the need for efficient SoW utilization by crops. By contrast, SW had the lowest conversion rate (6 %), which aligns with the observation that surface runoff is low in KD areas [49], and only a small part of the precipitation is converted to surface runoff [50]. In agroforestry and non-agroforestry areas, the conversion rate was respectively 6.27 % and 7.09 % for SW, 22.58 % and 23.10 % for GW, 35.67 % and 31.29 % for PW, and 63.03 % and 64.84 % for SoW. The results provide evidence that water-saving measures contribute to reducing biomass and vegetation interception, increasing the conversion of rainfall to SoW and GW, promoting five-water circulation, and realizing the efficient utilization of water resources.

## 4 Discussion

### 4.1 LE in karst area is equivalent to soil evaporation

**Fig. 7:**
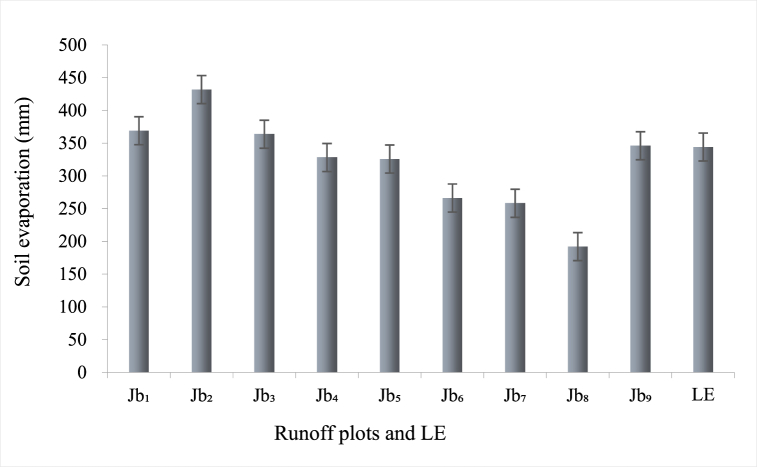
Soil evaporation and LE in Bijie study area. (Note: In this figure, Jb1-9 refers to the 9 runoff plots in Bijie, and LE is land evapotranspiration.)

LE in Koichiro Takahashi's formula is also known as evapotranspiration [51,52], land evaporation [53], and evaporation [54,55]. The formula includes two variables, rainfall and temperature. LE first excludes land evaporation and vegetation information, so it is a more appropriate term for soil evaporation, especially for ineffective evaporation of soil that does not include plant transpiration. Field measurements in our study showed that the sum of soil evaporation and plant transpiration was much higher than LE. Taking the Bijie research area as an example, in 2019, the total LE from April to August was measured as 344.13 mm, while a total of 587.06 mm was calculated by summing soil evaporation and vegetation transpiration, which is about 1.71 times higher. Soil evaporation in Jb_1_, Jb_3_, Jb_4_, Jb_5_, and Jb_9_ in this area was close to LE (Fig. 7). A similar trend was also found in the other two research areas, suggesting the reasonable argument that the LE in karst areas can be approximately seen as soil evaporation.

### 4.2 Improvement of crop water use efciency (WUE) by agroforestry

Crop water consumption is of great significance to agricultural WUE [27]. WUE is used to assess the ratio of plant production to water consumed [56,57]. It is also an important parameter for evaluating ecosystem water [58,59]. Drought often has a negative effect on WUE, yet moderate drought stress promotes the improvement of WUE [48]. In addition, different vegetation types significantly influence WUE. WUE of farmland ecosystems depends on evapotranspiration [[60], [61], [62], [63]]. Compared with monoculture crops, agroforestry increases the surface coverage, effectively reduces soil evaporation, and lowers the inner canopy temperature through shading [64]. Accordingly, it can reduce plant transpiration [65,66], inhibit ineffective water consumption, and promote the WUE of crops [24]. In addition, agroforestry has the advantage that most crops are planted on the smallest amount of land, which increases the crops and makes them more economically profitable. Given that karst regions are rainfed agricultural regions, agroforestry shows its superiority in the form of high WUE and the ability to achieve more economic benefits while consuming the same amount of water.

## 5 Conclusion

(1) Agroforestry reduced surface runoff while increased soil infiltration and GW, thereby reaching the ecological benefits of soil and water conservation.
(2) Since the larger amounts of biomass of agroforestry led to faster transpiration and larger vegetation interception, it is suggested to take water-saving measures such as dwarfing, dense planting, and pruning to increase the available precipitation, thus improving the efficiency of water-resource utilization in agroforestry.
(3) Soil evaporation in karst areas can be approximately seen as land evaporation (LE).
(4) In the process of precipitation transforming into the other four types of water, the conversion rate of SoW was the highest, followed by PW and GW, which is benefit to water resource utilization.
(5) Agroforestry systems, compared with non-agroforestry mode, contribute to more PW, GW, and SoW. Additionally, to cut down water consumption by plants and to promote more occurrence and transformation of GW and SoW, water-saving measures are necessary in improving WUE under agroforestry.

## Data availability statement

The data that support the findings of this study are available from the corresponding author, [Qinglin Wu], upon reasonable request.

## CRediT authorship contribution statement

**Qinglin Wu:** Writing – review & editing, Writing – original draft, Software, Resources, Project administration, Methodology, Investigation, Funding acquisition, Formal analysis, Data curation, Conceptualization. **Lan Wang:** Writing – review & editing, Visualization, Project administration, Investigation, Funding acquisition.

## Declaration of competing interest

The authors declare that they have no known competing financial interests or personal relationships that could have appeared to influence the work reported in this paper.
